# Accessibility, clarity, and organizational opportunities to enhance interprofessional collaboration in alcohol interventions: A qualitative study

**DOI:** 10.1186/s12913-025-13552-5

**Published:** 2025-10-21

**Authors:** Ruud T. J. Roodbeen, Jeroen Bruinsma, Andrea D. Rozema, Rik Crutzen, Sarah E. Stutterheim

**Affiliations:** 1 Department of Health Promotion, Care and Public Health Research Institute (CAPHRI), Maastricht University, Maastricht, the Netherlands; 2 IrisZorg, Institute for Addiction Care and Sheltered Housing, Research Department, Arnhem, the Netherlands; 3 Nijmegen Institute for Scientist-Practitioners in Addiction (NISPA), Radboud University, Nijmegen, the Netherlands; 4 Department Tranzo, Academic Collaborative Centre Addiction, Tilburg University, Tilburg, the Netherlands

**Keywords:** Interprofessional collaboration, Semi-structured interviews, Thematic analysis, Healthcare providers, Social workers, Problematic drinking

## Abstract

**Background:**

The implementation of effective interventions targeting problematic drinking in healthcare and social care remains limited, leading to fewer people changing their problematic drinking patterns. Improving interprofessional collaboration could enhance implementation of interventions targeting problematic drinking, yet a comprehensive understanding of interprofessional collaboration in the Netherlands regarding these interventions is currently lacking. This study set out to identify perceptions and experiences with interprofessional collaboration and the implementation of interventions targeting problematic drinking among professionals working in healthcare, social care and public sectors in a region of the Netherlands where problematic drinking is a known challenge.

**Methods:**

Guided by a phenomenological perspective, we conducted a cross-sectional interview study with 22 professionals. Semi-structured interviews were recorded and transcripts were analysed for themes using Atlas.ti.

**Results:**

Participants described their roles in interprofessional collaboration as minimalists (i.e., working strictly task oriented), brokers, or skilled networkers depending on their function and their perception of task and role fulfilment as a professional. Accessibility, clear role definitions, and leadership were identified as key to initiating and maintaining interprofessional collaboration. Challenges in interprofessional collaboration included regional, financial, or professional boundaries. Additionally, participants who were insiders of regional collaboration structures found these structures beneficial to interprofessional collaboration, while outsiders perceived them to be an impediment to collaboration and information exchange.

**Conclusions:**

Interprofessional collaboration structures seem to enhance collaboration among professionals, and, as a result, enhance the implementation of interventions targeting problematic drinking. Management of healthcare, social care, or public sector organizations should recognize that outsider-organizations may have limited access to these structures. They should therefore appoint network-oriented professionals to act as brokers, linking external organizations to enhance collaboration and information exchange. Additionally, organizations should provide flexible scheduling for their professionals so that they can concentrate on tasks beyond their immediate responsibilities, which will contribute to interprofessional collaboration and early detection of problematic drinking. Lastly, further research on intervention implementation should focus on expertise-development within teams to improve interprofessional collaboration, and consider how the perspectives of populations at risk for problematic drinking can be incorporated into collaboration-structures.

**Supplementary Information:**

The online version contains supplementary material available at 10.1186/s12913-025-13552-5.

## Background

Implementation of interventions targeting problematic drinking necessitates interprofessional collaboration. Interventions targeting problematic drinking in this study are defined as structured evidence-based approaches aimed at reducing problematic drinking and its associated risks through methods such as screening, brief interventions, behavioural counselling, and referrals to specialized care, often tailored to individual needs and integrated into healthcare or community settings [1]. Interprofessional collaboration is defined as a dynamic and integrated teamwork process by which different healthcare and social care professionals work together integrating expertise across professional boundaries to improve professional practice and healthcare outcomes, grounded in shared decision-making, shared responsibilities, mutual respect, communication, and understanding of each other’s roles [2]. Problematic drinking in this study is defined as a pattern of alcohol consumption leading to adverse outcomes, including impaired health, social issues, or behavioural consequences [3].

### Interventions targeting problematic drinking

Despite studies demonstrating the effectiveness of interventions targeting problematic drinking [1, 4, 5], and World Health Organization’s (WHO) recommendation to provide effective brief interventions through prevention, treatment and care [6], implementation of such interventions is limited [7–9]. Due to limited implementation, the availability of interventions targeting problematic drinking in social and healthcare settings remains hampered [7–9], making it difficult for people to change their drinking behaviour.

### Strategies to enhance implementation of interventions targeting problematic drinking

Three strategies to enhance implementation of interventions targeting problematic drinking by organizations and professionals have been suggested: (1) integrate interventions targeting problematic drinking into routine care [9–11], (2) enhance awareness and expertise on interventions targeting problematic drinking among professionals, considering existing staff attitudes and organizational culture [5, 7, 8], and (3) enhance inter- and multidisciplinary collaboration [2]. While numerous studies emphasize the importance of interprofessional collaboration between various actors during and after general implementation processes [2, 12–15], this strategy has received limited attention in research focused on interventions targeting problematic drinking in health and social care settings.

### Earlier studies on interprofessional collaboration for interventions targeting problematic drinking

To the best of our knowledge, there are two recent studies that have focused on interprofessional collaboration related to the implementation of interventions targeting problematic drinking in healthcare (e.g., general practices in primary care or academic medical centres). The first is a meta-analysis conducted by Keurhorst et al. on controlled trials [16]. This meta-analysis demonstrated that interprofessional collaboration is critical for embedding interventions targeting problematic drinking in primary care, and emphasized the need for teamwork, clear roles, shared training, and better integration into everyday care processes. The second is a focus group study by Hodgson et al. that explored barriers and facilitators to implementing SBIRT-strategies (Screening, Brief Intervention, and Referral to Treatment) in both academic medical centres and primary care clinics [17]. They highlighted a lack of clear communication and role clarity between different disciplines as a key challenge directly affecting team collaboration. Also, they emphasized that team structure, leadership, and shared motivation are essential for successful SBIRT implementation, and these are all central to interprofessional collaborative practices.

These studies have shown that interprofessional collaboration in primary care and academic medical centres can support implementation of interventions targeting problematic drinking. However, in addition to primary care and academic medical centres, various other sectors are also engaged in addressing issues related to problematic drinking. Further research should include social care professionals (e.g., working in community health services or home care) and professionals working in public sectors (e.g., police, housing corporations, municipalities) to enhance insights. This is essential because problematic drinking encompasses multiple facets beyond medical issues. It often intersects with social challenges such as unemployment and homelessness across various contexts, including home, workplace, and public settings [18]. To gain a comprehensive understanding of interprofessional collaboration for interventions targeting problematic drinking, it is necessary to also gather insights from these sectors. Additionally, previous studies have indicated that social care professionals are expected to engage proactively in interventions addressing problematic drinking [19, 20]. However, these social care professionals encountered barriers to doing so, highlighting the need to include their perspectives. This can contribute to more effective implementation.

### This present study

This study set out to identify perceptions and experiences with interprofessional collaboration and the implementation of interventions targeting problematic drinking among professionals working in healthcare, social care and the public sector in the Netherlands. This is important because enhancing the implementation of interventions targeting problematic drinking is likely to result in better accessibility [7–9], enhance effectiveness of interventions targeting problematic drinking [1, 4–6], and in turn, reduce problematic drinking.

## Methods

### Study design and context

This cross-sectional qualitative study is part of broader project, namely ‘Vital & (G)old’, whose aim is to develop an integrated approach to alcohol interventions for older adults. Initiated in 2021 and concluding in 2026, the Vital & (G)old project is being implemented in a region located in the South of the Netherlands. This area was selected based on its elevated rates of substance use as well as its demonstrated capacity and support for effectively addressing these challenges [21–23]. In addition to this qualitative study, a social network analysis (SNA) is in progress in the Vital & (G)old project. The primary purpose of the SNA is to describe and assess interprofessional collaboration among organizations in the South of the Netherlands that are involved in interventions changing problematic drinking patterns. Both the SNA and this qualitative study investigate interprofessional collaboration, focus on interventions changing problematic drinking patterns, and are conducted within the same region in the South of the Netherlands. While the SNA employs a quantitative methodology to describe the structure of the network, this qualitative study explores professionals’ perceptions and experiences within the network.

This qualitative study employed a phenomenological approach, which focuses on describing meaning and significance of experiences [24, 25]. A phenomenological approach enables participants to express their experiences and perceptions on cooperating with network contacts using their own words [24, 25]. The COREQ-checklist was followed in reporting, as this checklist is developed for qualitative studies that use in-depth interviews and focus groups [26].

### Sampling and recruitment

In the SNA study that preceded this qualitative study, participants were asked to identify contacts related to alcohol interventions for older adults and if they would be willing to engage in an interview (i.e., voluntary response sampling). If they were willing, they provided consent to receive information about the interview and provided their contact details. Participants that consented to being contacted (*n* = 38) were then emailed with information about the qualitative study and invited to participate. Of those 38, 22 were interviewed. The 16 participants that were not interviewed provided the following reasons for non-participation: lack of interest in participating in an interview, lack of interest in the topic, limited time for an interview, limited perceived expertise, or no response.

Table 1 shows participant characteristics for the 22 participants, 19 of whom were women. Most had a college or university degree, worked as home counsellors or mental health practitioners in general practice, and worked for community care/social support organizations. All were employed the study region in the South of the Netherlands. Five participants worked in the community support sector (e.g., social support and reintegration organizations), and five in secondary care sectors (e.g., addiction facilities or hospitals). There were four participants in each of the remaining sectors: primary care (e.g., general practices), other care organizations (e.g., home care organizations), and other non-care organizations (e.g., police or housing corporations).

Nearly half of the participants in the qualitative study reported not having encountered interventions targeting problematic drinking in their occupation when they completed the questionnaire for the SNA. Furthermore, none of the participants reported knowledge of, or involvement in, partnerships for interventions targeting problematic drinking or problematic drinking in general (part 3 of the topic list; Appendix 1). When this was confirmed by the participants during the interviews, the focus of the questions shifted from these specific interventions targeting problematic drinking to their general perspectives on interprofessional collaboration and problematic drinking. Additionally, most participants indicated that problematic drinking was not their main focus in their daily work. However, participants described this behaviour as connected to the aspects of their work and their clients’ behaviours.

**Table 1:** Participant characteristics (*n* = 22*)

Gender	
Female	19
Male	3
Average age (SD; min-max)*	50 (11; 27–64)
Education level*	
Less than high school	0
Vocational training	4
College or University	16
Occupation	
Home counsellor	3
Mental health practitioner in general practice	3
Housing corporation/energy consultant	2
Outpatient counsellor	2
Social psychiatric nurse	2
Behavioural scientist	1
Expert by experience	1
General practitioner	1
Informal care consultant	1
Lifestyle coach	1
Local police officer	1
Process supervisor	1
Psychologist	1
Social worker	1
Triage nurse	1
Organization in which functions were conducted	
Community care/social support organization	5
Addiction/mental health care organization	4
General practice	4
Home care organization	4
General hospital	1
Housing corporation	1
Municipality	1
Police	1
Safe at home organization	1
Sectors in which functions were conducted	
Community/social	5
Primary care	4
Secondary care	5
Other care organizations	4
Other non-care organizations	4
Participants coming across interventions in their function	
No interventions	9
Between 1 and 2 interventions	11
Between 2 and 4 interventions	1
More than 4 interventions	1

* two interviewed participants were invited for the questionnaire, but did not fill out the questionnaire, hence two missings in indicated variables

### Data collection

The one-on-one interviews were semi-structured and guided by a topic list. An initial version of the topic list was developed by the researcher (RR) based on literature on interprofessional collaboration and experience from previous research interviewing healthcare professionals (e.g [27, 28]). Feedback on this initial version was provided (AR, SS, RC), and after some minor adjustments, the topic list was completed (RR; Appendix 1).

Interviews were conducted through Microsoft Teams (20 times), by telephone (1 time), or in-person (1 time) at the workplace of participants, in accordance with participants’ preferences. All interviews were conducted by one researcher (RR), who was trained and experienced in qualitative research. During all interviews, only the researcher and participant were present. All interviews were carried out between July of 2023 and March of 2024. Participants verbally agreed to the informed consent form at the beginning of the interview. All interviews were audio-recorded and transcribed verbatim by an external transcription service. Interview duration was on average 51 min, ranging from 32 to 66 min. After each interview, the researcher (RR) wrote brief field notes to summarize key findings and general observations. One of the participants requested a member-check on the transcript of the interview. This was approved and facilitated by the researcher (RR). After checking, the participant accepted the transcript without providing feedback.

### Data processing and analyses

Transcripts and field notes were processed in Atlas.ti [29] and two researchers (RR & JB) conducted thematic analysis in accordance with Braun and Clarke [30, 31]. They first independently read and coded five transcripts and field notes to generate, discuss, and review initial and preliminarily codes. After individually reading and coding a transcript, the researchers held four meetings. During these meetings, they discussed and reached consensus on the initial codes and developed a preliminary framework of themes and subthemes derived from the data. Axial coding was applied in this phase to name, position and describe preliminary (sub)themes. A coding scheme (Table 2) was developed to present themes and subthemes, serving as a guide during meetings between the two researchers (RR & JB). This process and preliminary findings were reviewed by the principal investigator (SS). Following consensus on the initial codes, themes, and subthemes, one researcher (RR) independently coded the remaining transcripts and field notes, employing axial coding to complete the analysis.

All (sub)themes that were generated during the thematic analysis were illustrated by multiple quotes in the results section of this study. Quotes were numbered and labelled with the participant’s profession (Table 3). These quotes were translated into English and edited, increasing readability without loss of meaning or context. To ensure transparency and accuracy in presenting the findings of this study, quotes of different participants were selected with a diverse range of professions. Additionally, this study consistently uses the term “clients” to refer to all descriptions of clients, patients, and residents when presenting results.

### Research team and reflexivity

A trained interviewer (RR, PhD) conducted all interviews and led the thematic analysis, while a second researcher (JB, PhD) independently coded the transcripts. Both are Dutch-speaking, cis-male postdoctoral researchers familiar with the study region and its dialect. They are trained in qualitative, in-depth semi-structured interviewing and qualitive analysis, and have extensive experience in healthcare and public-health research (e.g [28, 32]). No prior relationships existed between the researchers and the participants, and participants had no knowledge of the researchers before the study.

### Ethical considerations

To protect the privacy of participants, all audio-recordings were deleted after transcription, and all the information that could reveal the identity of the participants were deleted from the transcripts. Transcripts and personal information were stored securely on servers of Maastricht University. The study protocol was evaluated and approved by the Faculty of Health, Medicine, and Life Sciences Research Ethics Committee from Maastricht University (file number: FHML-REC/2023/044), indicating adherence of this study to the Declaration of Helsinki.

## Results

Below, we first describe the procedures and meetings attended by healthcare, social work, and public sector professionals within the study region. We then outline the cooperative structures in which these professionals engage. Subsequently, we describe the themes and sub-themes pertaining to interprofessional collaboration (Table 2). For each theme, we provide exemplary quotations. Each quotation is numbered and linked to the participant’s profession (Table 3).

**Table 2:** Themes and subthemes generated during analyses

Themes	Subthemes
1. Individual characteristics relating to cooperation	Being accessible, visible, and responsive
	Being honest, clear, and open (trusting each other)
	Being involved (with the profession, organisation and collaboration)
	Knowing about other professions and leadership
	Treating each other as equals in a safe and pleasant environment
2. Self-awareness in cooperation	Reflecting on roles and abilities
3. Selecting roles and responsibilities based on professional perspective	Delineating own roles and responsibilities in care and cooperation
	Expanding focus and interests (looking at underlying problems)
4. Organizational and environmental conditions for cooperation	Regional boundaries
	Financial boundaries
	Professional boundaries (patient-provider confidentiality)
	Tenders and consortia
	Interprofessional collaboration between sectors
	Facilitating and investing in networking opportunities

### Procedures and meetings between professionals in the region

#### Addressing clients

Participants outlined protocols for addressing clients with problematic drinking in the community/social sector. Generally, cases involving complaints or requests for assistance, financed by the municipality, are sent to the community care/social support organization. A triage nurse in the relevant neighbourhood examines the case, possibly visiting clients or consulting other professionals. In weekly neighbourhood meetings (NMs), the case is assigned to an outpatient counsellor, home counsellor, or social worker. Clients may also visit community centres for support groups facilitated by the organization, where they can discuss their needs and be referred to specialized care if needed.

Health insurance companies typically finance primary and secondary care sectors. Clients schedule appointments with general practitioners (GPs). For problematic drinking, the GP may: (1) treat the client, (2) refer to a mental health practitioner (MHP), (3) refer to specialized addiction/mental health care, (4) refer to prevention specialists, or (5) refer to the community/social sector. The MHP can also treat or refer the client further. In specialized care, clients undergo detoxification and treatment for addiction. Besides regular care financed by insurance and organized by the government, private clinic treatments are available. Clients can also register for meetings with prevention specialists to evaluate drinking behaviour and develop intervention plans. These specialists may refer clients back to the GP, to the community/social sector, or to specialized care if needed.

#### Cooperation among professionals

Participants described different collaboration and consultation structures based on specific cases (see Box 1 for an overview). Collaborations could be individual, group-based, or multidisciplinary. Besides internal meetings like NMs and peer assessment meetings, community/social sector professionals could arrange round-table meetings (RTMs) for complex cases needing professional alignment. Case directors initiated RTMs, with clients either attending or consenting to them, unlike NMs. All involved professionals, mainly from the community/social sector and sometimes MHPs, were invited to RTMs. These meetings helped to align involvement, planning, and communication among the professionals regarding a client.

Most participants in our study were aware of weekly multidisciplinary consultations (MDCs) organized in primary care within the region. These MDCs were initiated in primary care, unlike most MDCs that originate from secondary care. The MDCs focused on cases of clients requiring additional expertise or alignment from other care sectors. Clients did not attend MDCs, and the professionals involved varied based on the required expertise. Participants noted the presence of GPs, MHPs, specialized physicians, nurse specialists, therapists, and social workers. While problematic drinking was rarely the focal point of discussion during MDCs, the subject was mentioned in most cases discussed, according to participants.

Participants mentioned bi-weekly general case and liveability (i.e., habitability, how it feels to live somewhere) meetings (CLMs), organized by the municipality, involving care professionals, law enforcement, housing corporation staff, and community volunteers, among others. Clients did not attend these meetings. CLMs focused on neighbourhood safety and liveability issues, including problematic drinking, nuisance from addiction, polluted areas, loneliness, social isolation, and poverty. The aim was to share and discuss these topics, align tasks and responsibilities, and address or resolve the issues.

**Box 1:** Cooperation structures among professionals in the study region

**RTMs:** Round-table meetings. Initiated by case directors working in the community/social sector. Including all professionals from this sector, and sometimes MHPs from primary care. Clients attend or provide consent.	
**MDCs**: Multidisciplinary consultations. Initiated by GPs and/or MHPs working in primary care. Include professionals from all care or community/social sectors of which expertise is needed. Clients do not attend.	
**CLMs**: Case and liveability (i.e., habitability, how it feels to live somewhere) meetings. Initiated by the municipality of the study region. Including professionals from all sectors (also law enforcement). Only discussing general issues, clients do not attend.	

### Interprofessional cooperation

#### General assessment of meetings

Most participants generally viewed cooperation in the region and during meetings positively, highlighting the importance of collaboration and recognition by others (Quote 1; Table 3). They noted that attending meetings helps them to understand professionals’ roles and tasks, learn from different perspectives, keep cases at the forefront, and share concerns. Cooperation fosters closeness and prevents isolation, and joint decisions reduce the workload. Participants felt supported and satisfied when their advice was heard and implemented. They also mentioned that RTMs, MDCs, and CLMs help to save time by addressing complex issues early. While MDCs have enabled cooperation for decades according to participants, not all GPs in the study region attend or organize them.

**Table 3:** Participant’s profession and quotes

**Quote 1:** Municipal energy consultant	Participant: It is a collaboration (the participant is talking about the CLM) where, initially, we were determining what we could share with each other and how we could be of value to one another. The necessity for this collaboration is increasingly recognized, and it is important to work together. We often do not know what others are doing, despite our collective efforts. This meeting (the CLM) demonstrates that it does not matter which party you belong to; if you can contribute to improving the neighbourhood in any area, your participation is valuable.
**Quote 2**: Local police officer	Participant: It is important to have professional knowledge, understand your network partners, and work based on trust. Often, information is shared that is permitted under a covenant, but it can be unclear whether sharing certain details is allowed. There are distinctions between ‘nice to know’ and ‘need to know’ information. Trust among professionals is crucial for effective work and transparent information sharing, understanding what is permissible. Sometimes, it is necessary to state that no information can be shared due to an ongoing investigation. Transparency is essential in cooperation.
**Quote 3**: Social Psychiatric Nurse	Participant: How should I put this? Allow me to explain properly. The responsibility that home counsellors feel towards their clients, similar for all individuals working at home care organisations, is that they struggle with the existence of problems such as addictive behaviour. They often do not clearly understand where their responsibilities lie in these situations. They quickly feel accountable for their clients‘ behaviour in the care process, leading them to seek coordination with us more promptly. (…). And then, they expect us to provide a solution. However, we do not always have one readily available. In fact, often we do not.
**Quote 4**: Outpatient counsellor	Participant: I often question if I’m the right person for this case. I don’t think I am. Interviewer: Who should be in charge then? Participant: I believe it should be M…(an addition/mental health organization in the study region). The GP referred the client to M…, involving a psychiatrist, psychologist, and SPN (social psychiatric nurse), a whole team actually. After investigating the client, they found a neurocognitive basis (Korsakov) and then referred the client to a neurologist, and that’s it! But that was too simplistic. This case still requires more from me than other cases, and should be dealt with by others.
**Quote 5**: Mental health practitioner	Interviewer: When interacting with professionals from M… (an addition/mental health organization in the study region) during the MDC, what do you think is important? Participant: As the chair, I like to keep the atmosphere pleasant by making jokes. It helps everyone feel more invested and relaxed. Interviewer: Yes. Participant: It’s important to maintain a neat but enjoyable environment. This creates equality and safety, allowing professionals to freely share their thoughts without fear of ridicule or hierarchy. Interviewer: Yes, exactly. Participant: Professionals can express doubts openly and discuss clients without judgment.
**Quote 6**: Informal care consultant	Interviewer: How do you generally experience your contacts with other professionals? Participant: Without my network, I would feel isolated and unsupported in advocating for my capabilities. However, because I have a strong network with many contacts from former colleagues and professionals in various workplaces in the Limburg region (province in the South of the Netherlands), I can reach out via phone, messaging, or email for assistance or consultation. I can ask them for guidance on what to do next or to collaborate with me on certain matters. This network is crucial in providing the support and advice that I need.
**Quote 7**: Outpatient counsellor	Interviewer: Do you prefer to arrange things by phone or email? Participant: Phone and in person. I find texting and emailing less effective as my communication does not translate well through those mediums. Therefore, I prefer to call or visit. Especially when working with new clients who have significant issues and multiple stakeholders, I organize an RTM for everyone to understand each other’s roles. This approach is also used when collaborating with a GP, particularly for addiction cases, where direct communication with the GP and MHP is essential.
**Quote 8**: Housing corporation consultant	Interviewer: Your role from W… (housing corporation company in the study region) matters too, right? Participant: Yes, I always say you can drink as much as you want as long as you don’t cause any nuisance. It’s up to you to change your behaviour so we aren’t bothered by it. But it’s not my job to ask someone to drink less. My concern is that you keep quiet and don’t bother your neighbours. The drinking itself is not my problem. Interviewer: Okay, so that it’s not your role? Participant: We go into people’s houses because we’re the housing association. However, it’s up to them to choose to change their behaviour. They don’t have to change their drinking for me. That’s a different starting point. We have no role if there’s no underlying problem for us. As a housing association, I don’t have to interfere with those twenty cans of beer.
**Quote 9**: Triage nurse	Interviewer: What do you think is important in establishing good contact with other professionals or colleagues? Participant: It is very important that people think ‘out of the box’. While privacy, legislation, and regulations are crucial, it is also necessary to consider broader perspectives to assist people properly. With some organizations, privacy regulations can hinder collaboration, leading to concerns about whether individuals receive adequate help. For instance, at M… (an addiction/mental health organization in the study region), there was a situation where I was really seriously worried about someone, but we did not get permission for data exchange. This restriction sometimes makes it challenging to share or receive important information that could contribute to safety or effective intervention.
**Quote 10**: Psychologist	Participant: Securing a contract with the health insurer posed some challenges for us. L… (an addiction/mental health organization in the study region) informed us that they would like to collaborate, but could not, due to contractual obligations. Their contract stipulates that they cannot work with non-contracted care providers. So, the difficulty we faced was mainly due to the restrictions imposed by health insurer contracts. They explicitly stated: “As long as you are not contracted, you should not approach us.”
**Quote 11**: Psychologist	Participant: You simply cannot get involved (into the regional consortium). You simply cannot. There is no possibility to have a conversation with them at all. They have their own partners and won’t consider new ideas. I’d like to see more opportunities and better communication. They are stuck in their own bubble.
**Quote 12**: Outpatient counsellor	Interviewer: And that either suits you or it doesn’t, right? And if the latter is the case then, then it gets a bit complicated. Participant: We have a few of those people working here, who find it difficult (i.e., find networking difficult), but who are, for example, very good for clients who need very long-term-attention. You often have, for instance, elderly people with addiction problems, people for whom an improvement trajectory is no longer really possible. You need people who are good at that, with stamina. So, we do have a few of them in our team, but you shouldn’t put them on the very innovative networking-projects.

### Individual characteristics relating to cooperation

#### Being accessible, visible, and responsive

Participants emphasized that being accessible, visible, and responsive is crucial for cooperation. They valued short communication lines, personal connections with professionals, and having permanent contacts at organizations. Timely responses to emails, texts, or calls were perceived as important throughout client care. Additionally, participants noted the need to be actively present and involved in meetings. Participants noted also that time constraints hinder accessibility. They described GPs as hard to reach due to busy schedules, limiting their ability to attend RTMs, MDCs, or CLMs. Additionally, some professionals preferred to work alone and struggled to prioritize meetings. In some cases, community/social sector professionals did not know the GPs of clients, complicating communication.

#### Being honest, clear and open (trusting each other)

Honesty, clarity, and openness were crucial for effective collaboration. Participants emphasized the need for transparency about roles, capabilities, and expectations. They valued actions aligning with words, fulfilling promises, and being clear about limitations. This expectation extended to organizations as well, which participants said should be transparent about their non-profit status and operations. Participants valued open communication and attitudes during collaboration, describing it as being reflective, accommodative, and helpful in meetings. Timing was also considered crucial, especially in RTMs with clients who might need reassurance. Trust in expertise, honesty, and clarity were further considered essential for effective collaboration. Participants emphasized that expressing concerns should be seen as experience-based with no hidden agenda. They also highlighted the importance of trust when handling private client information (Quote 2).

#### Being involved (with the profession, organisation and collaboration)

Involvement was considered crucial for effective collaboration, according to participants. They noted that staying updated on new developments, having an affinity with the topic and client population, obtaining valuable information, and engaging in networking activities are essential. Weekly communication with peers and active involvement during meetings, including listening and asking questions, enhanced collaboration. Additionally, including other professionals in the decision-making process ensured they did not feel excluded, thereby fostering cooperation. Participants also highlighted that collaboration is facilitated by motivated and cooperative clients.

Some participants indicated wanting more involvement in the network. To achieve this, one participant suggested self-initiated work experience days with professionals from different organizations. Participants also highlighted the importance of showing GPs the benefits of organizing MDCs and getting them more involved. Active listening, showing involvement, and asking about the professionals’ own psychological well-being during collaborations were considered crucial for enhancing cooperation.

#### Knowing about other professions and leadership

Participants highlighted the importance of understanding each other’s roles, expectations, and leadership to effectively collaborate. This involves understanding the roles and expertise of different professionals and how they address problematic drinking (Quote 3 for an example). Several participants observed that professionals often lack a comprehensive understanding of their colleagues’ roles and expectations when managing complex cases of problematic drinking, resulting in impeded collaboration. Participants noted challenges in maintaining oversight of internal and external professionals with expertise in issues such as problematic drinking.

Additionally, participants noted that, in collaborative work, it is important for everyone, including clients, to be aware of the lead professional (or organization) responsible (Quote 4 for an example). This applied to both external and internal professionals. The lead professional should ensure all relevant details are aligned with other professionals before meeting clients to prevent any miscommunication and confusion regarding care planning or other care-related information. Particularly participants working in the community/social sector noted a lack of leadership, leading to clients being overlooked. In addition, they saw colleagues with heavy workloads taking on poorly led cases, increasing burnout risk. They recommended having clear leadership from the outset of client care, with transparency and control for both professionals and clients throughout the process.

#### Treating each other as equals in a safe and pleasant environment

During collaboration, participants indicated wanting an environment that is equal, safe, and pleasant, where everyone treats each other with respect and can freely express their thoughts and concerns. The MDC was frequently mentioned by participants as an example of such an environment. In addition to promoting cooperation and productivity, participants described MDCs as sociable and pleasant. One participant noted that the safe, sociable, and pleasant nature of the MDC is a key factor for achieving success (Quote 5).

### Self-awareness in cooperation

#### Reflecting on roles and abilities

Most participants viewed themselves as demand-driven professionals focused on their specific roles and tasks, collaborating only when necessary. They preferred having full schedules with clients, minimalizing cooperation. Some participants saw themselves as brokers, facilitating connections between organizations when necessary. These participants believed it was their responsibility to maintain a professional attitude towards interprofessional collaboration. A minority described themselves as experienced networkers who excel at building and maintaining professional relationships (Quote 6 for an example). They valued their extensive networks and were willing to share them with others.

Some participants elaborated on their own roles and abilities in networks, offering different viewpoints. Participants experiencing isolation from the regional consortium (see Theme 4) evaluated their perspectives on collaboration, concluding that their viewpoints might diverge from those of other professionals within the consortium, thereby emphasizing perceived barriers. Furthermore, participants also reflected on connecting with clients and other professionals within the limited time they have. One participant noted difficulties in clarifying to peer-professionals that they are not solely responsible for clients’ problematic drinking behaviour and that expectations for specialist care referrals are often too high, causing disappointment for both professional and client. This participant advised viewing their role as taking small steps in the clients’ recovery journey. Additionally, some participants adjusted their communication style based on self-awareness (i.e., reflecting on their own demeanour when communication trough texting and emailing instead of calling or visiting someone; Quote 7).

### Selecting roles and responsibilities based on professional perspective

#### Delineating own roles and responsibilities in care and cooperation

During interviews, participants were asked to explain their reasons for not using or referring to interventions targeting problematic drinking in their professional capacity, providing additional insights on why participants choose their role in interprofessional collaboration. Typically, participants based their reasoning on their professional roles and responsibilities. Essentially, if a client’s problematic drinking behaviour does not fall within the participant’s role or responsibilities, then it is not their duty to address it with the client, or discuss it in interprofessional collaboration. This delineation of roles and responsibilities was particularly evident among participants working at municipalities, housing corporations, police, or other non-healthcare organizations (Quote 8 for an example).

Similarly, some participants focused solely on the specific task or care request at hand, such as bandaging a client’s legs, despite noticing other health issues. Participants from the community/social sector stated that this approach is guided by organizational policies to increase efficiency. They emphasized respecting the client’s invitation for a specific purpose instead of patronizing them by addressing unrelated drinking behaviour, and engaging in interprofessional collaboration about this. Additionally, some participants noted that they do not refer to interventions targeting problematic drinking because the drinking behaviour of their clients is too severe for prevention or community/social sector actions. However, according to participants, this can result in clients being overlooked, deemed too complex for prevention and social care, yet too simple for secondary care, causing them to fall through the cracks.

#### Expanding focus and interests (looking at underlying problems)

Despite working in specific roles, and having certain responsibilities and tasks, a minority of participants indicated a willingness to expand their focus and interests during their work. They remained vigilant about problematic drinking and discussed these issues in meetings with colleagues (e.g., during NMs, MDCs or CLMs). Some believe this approach is essential for being a good professional and expect all other professionals to act similarly. Furthermore, according to some of the participants, non-care/social professionals are crucial for detecting early signs of problematic drinking because they observe multiple aspects of clients’ lives. One participant noted that clients may not reveal issues related to problematic drinking to their care providers, but such issues may be noticed by housing corporations or police.

Participants with broader or more relevant role descriptions (e.g., MHPs, social psychiatric nurses, addiction behavioural scientists, psychologists) tend to focus more on addressing problematic drinking and engaging in interprofessional collaboration. They explained that they frequently offer interventions to clients or refer clients to specialised treatment, and some advocated for examining underlying problems causing alcohol use, a concept they call ‘positive health.’ These participants also shared their expertise with other professionals and suggested psychoeducation to help others understand the underlying complexities of alcohol use. Moreover, they indicated believing that, regardless of specific roles, every professional’s goal is to look at clients holistically, listen to them, and help them improve their lives.

### Organizational and environmental conditions for cooperation

#### Regional boundaries

Participants indicated that different sectors have varying regional boundaries in which they focus their activities, privacy regulations, and interprofessional collaboration. For example, in the study region, the community/social sector is divided by the municipality into two main areas with designated teams for each neighbourhood. In primary care, GP areas are regulated nationally, ensuring GPs can reach clients within fifteen minutes during emergencies. Within these areas, GPs conduct their own activities, privacy regulations and interprofessional collaboration.

The variation in sector areas and related activities, privacy regulations, and interprofessional collaboration, were said to affect overall cohesion between sectors. Participants indicated that, as a result of this variation, identifying suitable professionals from different sectors to collaborate with requires substantial time and effort. Furthermore, MHP participants primarily expressed concerns that client referrals from unfamiliar areas are often impersonal and short-sighted. They observed that usually it is the most complex and challenging cases that are assigned to them. Furthermore, according to participants, organizing MDCs is challenging due to difficulty matching professionals with clients because of these regional boundaries. Also, participants frequently encountered challenges in sharing or acquiring client information with colleagues from other professional sectors. Notably, within the community/social sector, difficulties in obtaining client information from the secondary care sector were experienced, even in situations where the safety of others is at risk (Quote 9 for an example). Nevertheless, participants often found ways to share information without violating privacy regulations. For example, they reported not mentioning clients’ names and birth dates but discussing other relevant health and safety information. A more sustainable solution suggested was expanding the use of information-sharing agreements between sectors (e.g., under a covenant), which some organizations in the social care sector were already using at the time of the study. Overall, participants indicated that experience and network-knowledge can help to mitigate these issues.

#### Financial boundaries

Participants observed that professionals are also financed from various sources, such as municipalities, health insurance companies, government, or private organizations. This, in turn, affects resources, perspectives, and interests, which can make cooperation complex. One participant provided an example involving clients’ significant others. The participant described that significant others receive assistance from different professionals funded by various sources. This makes interprofessional collaboration challenging due to varying time commitments, a focus on individual behaviours, or an emphasis on the immediate significant other instead of the entire family.

Additionally, for interprofessional collaboration, organizations generally need to have a contract with health insurance companies. If there is no contract, regional organizations might decline cooperation. This has hindered collaboration between organizations (Quote 10 as an example).

#### Professional boundaries (patient-provider confidentiality)

In cases when domestic violence and child protection services, or “Dutch Safe at Home organizations (Veilig Thuis)” were involved (with problematic drinking being part of the problematic behaviour), participants indicated that professional boundaries obstructed cooperation. Healthcare professionals adhere to patient-provider confidentiality, but this must be lifted when Safe at Home organizations report a case, requiring cooperation and sharing of information. These participants suggested that healthcare professionals need more knowledge about Safe at Home organizations and their reporting procedures.

#### Tenders and consortia

A tender is a procedure where organizations are invited to provide a service or product by submitting a proposal. A consortium, a temporary partnership between organizations, was initiated by the municipality investigated to implement Dutch Social Support Law (WMO-care). This consortium represents a collaboration of social and care organizations that help citizens with health, lifestyle, financial and practical challenges [33]. It includes community care/social support organizations, addiction/mental health care organizations and home care organizations. This study involved participants from both within and outside this consortium, offering insights into cooperation barriers and needs.

Participants included in the consortium viewed the collaboration positively, noting clear contacts and productive consultation structures. However, they identified some barriers, such as time needed for cooperation to flourish due to differing organizational traditions. Additionally, some professionals experienced difficulties adjusting to changes, which extended the time required for effective collaboration. Furthermore, participants indicated that, when tenders or consortia change or disband, established contacts and consultation structures often disappear, hampering sustained collaboration.

Participants outside the consortium found the consortium to be isolated and inaccessible. One noted that consortia shut themselves off for new ideas, suggesting more openness to non-members (Quote 11). This participant also observed internal clustering within the consortium, making it even harder to have an overview of relevant expertise and to initiate collaboration to share ideas and insights on specific subjects.

#### Interprofessional collaboration between sectors

Participants identified several general barriers to interprofessional collaboration between sectors. First, participants reported that tensions often arise between GPs, versus the community/social sector and home care organizations due to differing opinions with respect to care responsibilities, especially when clients are unmotivated (i.e., home care organizations feel that more effort and care is needed from primary care). Second, though their presence is deemed crucial by participants, professionals in secondary care (particularly hospital staff), and prevention-specialists, rarely attend MDCs in primary care. Additionally, participants working in secondary care emphasized the importance of referring clients to the community/social sector, particularly when clients are stable enough for day-to-day activities. They suggested that, even after crises are managed, the community/social sector can resume care. These participants advocated for more education in the community/social sector: on interprofessional collaboration, on the early detection of addiction, on understanding underlying problems of addiction, and on knowing when to refer to secondary care.

#### Facilitating and investing in networking opportunities

Participants reported that networking and collaboration are ongoing iterative processes that require time, ongoing effort, and long-term investments. They indicated that networking and collaboration involve establishing relationships, mutual trust, and familiarity. Additionally, participants mentioned that results of networking and collaboration take time and require patience. Some participants felt that their supervisors in management often overlooked these points. Additionally, participants emphasized acknowledging the unpredictable nature of networking, such as unexpected changes in attendance of consultations or meetings by clients and/or professionals. It is also important for organizations to appoint competent professionals for networking tasks (Quote 12).

Participants listed various organizational and environmental needs to facilitate cooperation. They emphasized the need for more resources and time from their organizations to join and maintain networking opportunities. Participants also highlighted that it is important that their organizations focus on external and internal cooperation opportunities, such as organizing or attending MDCs or CLMs, which improve knowledge on roles, responsibilities, and care coordination. Additionally, participants called for increased regional cooperation and knowledge exchange, especially on interventions targeting problematic drinking and best practices on implementation.

### General needs and barriers

Several needs and barriers for cooperation in addressing problematic drinking were identified by participants. First, participants emphasized the importance of faster client allocation, especially in the community/social sector and secondary care. According to participants, delays demotivate both professionals and clients, and dilute existing cooperation-structures surrounding clients. Second, improved interaction between hospitals and other care sectors is necessary, as hospital staff are often unaware of developments elsewhere. Third, time constraints, especially for GPs, hinder cooperation. Fourth, more local meeting locations are needed in the community/social sector for client interactions and collaboration. Fifth, the current array of interventions targeting problematic drinking is seen as overwhelming and unclear, requiring more concise information. Finally, participants expressed the need for a comprehensive “social map” of professionals relevant to curbing problematic drinking, and referral guidelines available through existing digital tools. This should include a dedicated section on when and how to refer to prevention specialists.

## Discussion

This study set out to identify perceptions and experiences with interprofessional collaboration and the implementation of interventions targeting problematic drinking among professionals working in healthcare, social care and public sectors in a region of the Netherlands where problematic drinking is a known challenge. Participants provided insights into interprofessional collaboration among individuals, organizations, and sectors, identifying both hindering and facilitating factors. In summary, participants found interprofessional collaboration structures to be advantageous, particularly when they were actively involved and included within these frameworks. Additionally, participants described their roles in interprofessional collaboration as minimalists (i.e., working strictly task oriented, minimalizing cooperation), brokers, or skilled networkers, depending on their function and their perception of task and role fulfilment as a professional. Accessibility, clear role definitions, and leadership were identified as key to initiating and maintaining interprofessional collaboration. Challenges included regional, financial, or professional boundaries, and being an outsider of regional interprofessional collaboration structures.

Findings of this study indicated that participants engaged in interprofessional collaboration individually, or by participating in RTMs, MDCs, CLMs or regional consortiums. These collaboration-structures were seen as beneficial for interprofessional collaboration by participants. Similarly, earlier studies on interprofessional collaboration and the implementation of interventions targeting problematic drinking have identified structured teamwork and interprofessional meetings as beneficial for improving patient outcomes and implementation (e.g [2]). While most participants regarded these regional collaboration structures as advantageous (i.e., being insiders), a distinct nuance could be identified. Non-consortium participants found it isolated and inaccessible, with internal clustering further obstructing collaboration and the sharing of insights. This may reflect siloed communication, which is defined as the limited interaction or collaboration between distinct groups within social network structures [34, 35]. Siloed communication can create barriers to information flow, hinder coordination, and lead to inefficiencies in healthcare delivery [33, 34]. This concept seems to apply to experiences of participants in the current study and highlights a particular limitation of these interprofessional collaboration structures, thereby contributing to our theoretical understanding of these frameworks. Further research is needed to investigate the possibilities for increasing openness and collaboration within consortium structures, extending engagement to external organizations without regard to regional, financial, or professional boundaries. Professionals in organizations who view themselves as networkers (and especially these professionals, according to participants) could take on a broker role between the consortium and external organizations, and thus facilitate connections and the flow of information [36–39].

Participants in the current study highlighted the importance of understanding and clarifying roles, expectations, and leadership for effective collaboration. They also noted an absence of such understanding and clarity, particularly in the community/social sector. Earlier studies have shown that clear communication, role clarity, team structure, and leadership enhance interprofessional collaboration and implementation uptake [16, 17]. Additionally, earlier studies have shown that improving clarity in roles and responsibilities is necessary for health and social care professionals to provide coordinated, high-quality care during client trajectories and interprofessional collaboration [40, 41]. To improve clarity concerning roles, responsibilities, and leadership, previous research has highlighted the importance of cultivating relational and leadership skills within interprofessional teams, in which enhanced communication can lead to more coordinated and empathetic client care [42, 43]. As an implication for research, more insights are required to examine how expertise development in teams can improve clarity regarding roles, expectations, and leadership. Observational research that examines the expression of roles, expectations, and leadership of professionals during interprofessional meetings, such as RTMs, MDCs, or CLMs, may offer further contextual information relevant to the findings of this study and represents a possible direction for future research. Additionally, we recommend conducting action research that foreground participatory processes and co-creation with professional teams [44]. This could also improve our theoretical understanding of addressing leadership roles to enhance knowledge, change attitudes, and foster collaborative behaviour. This may include elements such as flexible leadership within interprofessional collaboration [45] or structured role clarification processes implemented early in interprofessional collaboration [40]. In addition to cultivating expertise within current teams, interprofessional education should also be emphasised during the foundational training of future health professionals. Prior research indicates that implementing interprofessional education exercises at early stages of provider education positively impacts participants’ attitudes and self-confidence [46]. Furthermore, ongoing education in this area, supported by robust accreditation processes designed to facilitate interprofessional opportunities—unlike in the current system in the Netherlands—is important for maintaining professional engagement in interprofessional collaboration.

The findings of the current study also provide implications for practice, focussing on management in healthcare, social care and public sectors to enhance interprofessional collaboration. To improve interprofessional collaboration, and in addition to more expertise development as suggested above, management may consider allowing professionals more time-flexibility in their work and move beyond solely focusing on tasks. This could enable professionals to take on roles as brokers or networkers during their work, rather than just being minimalists in interprofessional collaboration. Providing increased time-flexibility may also facilitate earlier identification of problematic drinking. Professionals are important in this context due to their regular interactions with people across different stages of life and health statuses. This early detection may help in preventing the escalation of alcohol-related issues and facilitate timely intervention [47, 48].

### Strengths and limitations

A key strength of this study lies in its in-depth qualitative design, which allowed for rich, contextualized insights into the dynamics of interprofessional collaboration across healthcare, social care, and public sectors. Semi-structured interviewing allowed for participants to articulate their experiences and perceptions in their own words, capturing valuable nuance. The studies’ diverse sample of participants from multiple sectors enhanced breadth and credibility of findings. Additionally, some limitations are to consider. First, there is potential for self-selection bias. Professionals who consented to participate had previously completed the questionnaire for the SNA, and may already suggest a higher level of interest in collaborative practice. Their perspectives may thus not represent those of less interested professionals. Second, this study concentrated on professionals’ perspectives regarding interprofessional collaboration, omitting the views of clients. Perspectives of clients are important for attaining a comprehensive understanding of how interprofessional collaboration could address their needs effectively. As an implication for research, we suggest investigating if, how, and when clients would prefer to participate in interprofessional collaboration. Future research should also prioritize populations at risk for problematic drinking, such as older adults. These groups have diverse care needs that extend beyond the scope and capabilities of individual sectors, requiring not only medical intervention, but also social support, housing stability, mental health care, and other services. This necessitates interprofessional collaboration, and investigating the perspectives of clients on such collaboration would be a fruitful avenue for future research (e.g [49]). Third, the findings are shaped by specific organizational and cultural contexts (e.g., the regional consortium in the study region) [33], potentially limiting transferability to other regions or health systems with different structural or policy environments. To gain a more comprehensive understanding of interprofessional collaboration, future research should investigate collaboration in other regions as well. Fourth, nearly half of the participants reported not having encountered interventions changing problematic drinking, and none reported involvement in partnerships for interventions changing problematic drinking. This may constrain the depth and transferability of the study’s findings. There might be some heterogeneity in domain knowledge among participants, and as a result, some of the participants may have provided less depth in their descriptions. This may have resulted in an underestimation of potential bottlenecks or solution strategies, thereby restricting the transferability of the findings to contexts where intervention maturity is more advanced. At the same time, the limited familiarity with interventions reflects a realistic starting point in many practices. By including different levels of experience, we map both perceptions and needs in early adoption and effective patterns in more experienced teams. This increases the practical usefulness of our recommendations. Fifth, our study primarily focused on interprofessional collaboration for interventions targeting problematic drinking. Future research exploring how to enhance the implementation of interventions for other substances should use insights from this study as a foundation and apply findings to other contexts.

## Conclusion

The study findings provide valuable insights into the perceptions and experiences of professionals with interprofessional collaboration and the implementation of interventions targeting problematic drinking. Interprofessional collaboration structures seem to enhance collaboration among professionals, and, as a result, seem to enhance the implementation of interventions targeting problematic drinking. Management of healthcare, social care, and public sector organizations should recognize that outsider-organizations may have limited access to these structures, and appoint network-oriented professionals to act as brokers, linking external organizations to enhance collaboration and information exchange. Organizations should also offer flexible scheduling to enable professionals to focus on tasks beyond their immediate duties, promoting interprofessional collaboration. For municipalities, municipal health services, and prevention specialists involved in deploying interventions in the Netherlands, the results of this study can serve as a background document to identify factors in interprofessional collaboration that may hinder or facilitate the implementation of interventions targeting problematic drinking. To curb problematic drinking more effectively, further research on intervention implementation should focus on expertise-development within teams to improve interprofessional collaboration, and consider how to incorporate the perspectives of populations at risk for problematic drinking into collaboration-structures.

## Supplementary Information

Below is the link to the electronic supplementary material.

Supplementary Material 1

## Data Availability

Pseudonymized interview transcripts are available upon request from the corresponding author, provided participants have consented to sharing for open science purposes.
