# Supplementary material for: Accessibility, clarity, and organizational opportunities to enhance interprofessional collaboration in alcohol interventions: A qualitative study
Source: BMC Health Serv Res. 2025 Oct 21;25:1384. doi: 10.1186/s12913-025-13552-5 (PMC12541934; doi:10.1186/s12913-025-13552-5)
Supplement: Supplementary file 1 — Supplementary Material 1 [file 12913_2025_13552_MOESM1_ESM.docx]

# Appendix 1 – Interview Guide

**Introduction**

In the questionnaire we previously asked you about your contacts with organizations and professionals related to alcohol interventions for the elderly. We also asked you in the questionnaire how often you have contact with these professionals and what you think of these contacts. With this information, we have been able to create a network that gives us important insights into the ways in which people from Heerlen-Noord collaborate. Thanks again for this!

In this interview we would like to delve deeper into your general experiences with the network in Heerlen-Noord. For example, we will ask you what you find important, or what you currently think is missing. All these questions are related to alcohol interventions for the elderly. In addition, in this interview, we would also like to present you with a schematic representation of the network that we created from the questionnaire data. We will ask you your general thoughts on this, and we will ask you to provide possible causes for results from this network. Do you have any questions about this so far?

**Part 1 – alcohol interventions for the elderly and general contacts/collaborations/partnerships**

- Can you briefly indicate how you deal with alcohol interventions for the elderly in your daily work?

- And in which function do you deal with this in most cases?

- Generally speaking, what do you think of these interventions?

- With which organizations do you sometimes have contact about this?

- If you have contact with professionals about alcohol interventions for the elderly:

o What are these contacts about in most cases?

o What was the reason for contact in these cases (e.g. more information, or referrals, or collaborations)?

o How did the contact proceed in these cases (e.g. via email or verbally)?

o Why did you choose this method of communication?

- Does your organization have a policy for entering into/dealing with these contacts? And do you comply to this policy? Why/why not?

- And how do your colleagues do that? Do they comply to this policy?

- In which collaborations and/or partnerships related to alcohol interventions for the elderly are you involved?

o How did the contact in these collaborations proceed (e.g. via email or verbally)?

o Why this way of communicating?

o Does your organization have a policy for these collaborations? And do you comply to that policy? Why?

o And how do your colleagues do that? Do they comply to this policy?

o And if you refer?

- Does your organization have a policy on that?

- Do you comply to that policy (i.e., what is common practice)?

- How do your colleagues do that?

**Part 2 – perceptions and experiences with contacts**

- What do you think is important to establish good contact?

- And to maintain contact?

- What role does the way of communicating play in this?

- What are your own experiences with the contacts that you have related to alcohol interventions for the elderly?

o To what extent are you satisfied with this?

- Why/why not?

o What facilitates you with this?

- Can you give an example?

o What makes it difficult?

- Can you give an example?

- How do you think this could be solved?

*Elaborate on levels below when missing in answers:*

1) *Intra-personal*, to what extent do you (as an individual) play a role in this?

- What do you need yourself?

2) *Interpersonal*, to what extent does your direct environment (colleagues) play a role in this?

- What do they need in your opinion?

3) *Agencies/organizations*, to what extent do agencies/organizations play a role in this?

- What role does your own agency/organization play?

- What role does your management play in this?

- What do they need in your opinion?

4) *Social/policy*, to what extent does society play a role in this?

- What role does national politics play?

**Part 3 – perceptions and experiences with partnerships**

- What do you think is important to create good partnerships?

- And to maintain good collaboration?

- What role does the way of communicating play in this?

- What are your own experiences with the partnerships that you have related to alcohol interventions for the elderly?

o To what extent are you satisfied with this?

- Why/why not?

o What facilitates you with this?

- Can you give an example?

o What makes it difficult?

- Can you give an example?

- How do you think this could be solved?

*Elaborate on abovementioned levels when missing in answers.*

**Part 4 – the network**

*[present schematic representation of network]*

*Here you see a schematic representation of the network that we created from the questionnaire data.*

*[briefly explain the schematic representation]*

- What does this evoke in you?

- What do you think of this network?

- Can you name connections that you did not expect?

- Can you name connections that you think are missing, but that you did expect?

- Can you name connections that you think we overlooked?

- Can you explain how your own network helps you in your work?

**Closing**

This was my last question. Do you have any questions, or anything to add? Do you feel like I have missed something about contacts, collaborations or partnerships regarding alcohol interventions for the elderly?

Thank you very much for your cooperation!
